# Psycometric Validation of an Instrument to Measure Person-Centred Teamwork in Hospital Settings

**DOI:** 10.1155/nrp/2185757

**Published:** 2025-04-12

**Authors:** Alida Viljoen, Ronell Leech, Paul Slater, Andries Masenge, Tanya Heyns

**Affiliations:** ^1^Department of Nursing Science, Faculty of Health Sciences, University of Pretoria, Pretoria, South Africa; ^2^Institute of Nursing and Health Research, Ulster University, Northern Ireland, UK; ^3^Faculty of Natural and Agricultural Sciences, University of Pretoria, Pretoria, South Africa

**Keywords:** empirical research, instrument, measurement, person centred, teamwork, validation

## Abstract

**Aim:** To validate an instrument for measuring healthcare workers' perceptions of person-centred teamwork in hospital units.

**Design:** Quantitative cross-sectional descriptive design. This approach collected numerical data to explore and describe the characteristics of the instrument items, with the goal of generating insights as to the validity and reliability of the items.

**Methods:** The target population included healthcare workers who worked in hospital settings. Total sampling was used to identify healthcare workers. Convenience sampling was used to select the participants. The participants completed the instrument. The data were captured and analysed using the software IBM SPSS Statistics Version 28 and RStudio 2023.06.

**Results:** A 38-item instrument measuring the perceptions of healthcare workers of person-centred teamwork was tested psychometrically. A total of 388 healthcare workers working in private (*n* = 160) and public (*n* = 228) hospitals completed the instrument. Confirmatory factor analysis was used, indicating that the items were significant and that the constructs were well measured. Factor loading was present, and bifactor analysis confirmed the multidimensionality of each construct. The Cronbach's *α* confirmed the reliability of each of the 38 items.

**Conclusion:** The person-centred teamwork instrument was reliable and validated as a multidimensional scale comprising 38 items. The instrument is psychometrically suitable for measuring person-centred teamwork in hospital settings.

**Implications:** The person-centred teamwork instrument provides the ability to measure person-centred teamwork efforts to improve practice. As a measurable concept, person-centred teamwork can be improved by distinguishing areas for improvement.

**Link to Practice:** The 38 item person-centred teamwork instrument indicated good fit for measuring the constructs, and the instrument was validated. Each of the items was reliable for measuring person-centred teamwork. The instrument can be applied internationally to assist in the measurement of person-centred teamwork practices to improve clinical outcomes.

## 1. Introduction and Background

The concepts of person-centredness and teamwork are two concepts that are embedded in healthcare and are both associated with improved patient outcomes [1–3]. In healthcare, person-centredness encapsulates all people involved in the healthcare process including patients, members of the healthcare team, significant others and community members [2]. Person-centredness involves thinking about people within an environment and creating a culture of trust, respect and mutual goals [2]. Similarly, effective teamwork creates an environment where the workload is shared and normally overwhelming tasks become more manageable. When teamwork is effective, team members share a sense of belonging, interact positively and experience job satisfaction, staff productivity, staff retention and deliver high quality care [2, 4, 5]. Effective teamwork is associated with improved job satisfaction and staff retention, which leads to better continuity of care and also contributes to improved patient satisfaction and patient outcomes [6, 7]. Teamwork is essential for successful person-centredness as it allows the multidisciplinary team members, patients and community members to share in the care process [8].

Person-centredness and teamwork are well developed and established concepts for improving practice and outcomes [2, 9]. Person-centredness demands that healthcare workers adjust their emphasis from the “disease within the person” to the “person with the disease” [10]. Healthcare teamwork focuses on interprofessional collaboration, with patient outcomes serving as a goal [11].

The interrelated use of person-centredness and teamwork has led to the defining and constructing of “person-centred teamwork” as a measurable concept in recent years. Person-centred teamwork as a single concept has been analysed and defined recently [12]. Separate measurable instruments exist for person-centredness [13, 14] and teamwork [15, 16], but there is a need to develop an instrument to measure the concept as a whole.

Developing an instrument requires multiple steps and rigour to obtain the validation and reliability needed to ensure that the items measure the concept accurately [17]. The scale was developed by pretesting the items, sampling and survey administration, item reduction and factor extraction [17]. An instrument has been developed to measure person-centred teamwork but has not been validated for its applicability in healthcare settings. To address the lack of validation, this study aimed to validate an instrument that was developed to measure person-centred teamwork in healthcare settings [18]. The research question asked was “Is the instrument to measure healthcare workers perceptions of person-centred teamwork in hospital units, valid and reliable?”

## 2. Methodology

### 2.1. Aim

To validate an instrument to measure person-centred teamwork in hospital units.

### 2.2. Design

A quantitative cross-sectional descriptive design was used to test the measurement of the instrument. This approach collected numerical data to explore and describe the characteristics of the instrument items, with the goal of generating insights as to the validity and reliability of the items.

### 2.3. Setting

The instrument was validated in the Gauteng province in South Africa, which is positioned on the southern tip of the African continent. South Africa covers 1.2 million km^2^ and has a population of 60.6 million [19, p. 18]. South Africa is recorded as a third world or developing country with high unemployment and poverty rates even though it has an abundance of goods and natural resources and is recognized as one of the largest industrialized countries in Africa in terms of both wealth and gross domestic product [20]. Furthermore, South Africa is divided into nine provinces [19, p. 18]. Despite being the geographically smallest, Gauteng Province has the largest population of the nine provinces, with just over 14 million inhabitants [21].

The South African healthcare system is currently a pluralistic system with separate public and private sectors, and third and first world health conditions are found in the population [22]. The public sector is funded by the National Treasury and Government. The estimated total healthcare expenditure in public sector hospitals is R122 bn annually. The private sector is compensated by medical schemes (medical insurance), which are funded by their members. The average annual expenditure in the private sector is R142bn [23].

For the purpose of validating the instrument, one public tertiary hospital in the city of Pretoria and one private hospital in Pretoria were selected. Convenience sampling was used. The public hospital was chosen due to its academic affiliation with the researcher's higher education institution. It is also in close proximity to the researcher's place of work. The private hospital was selected because it is the workplace of the researcher. Therefore, the data collection was more convenient. At the time of the study, a total of 987 and 343 healthcare workers were selected from public and private hospitals, respectively.

### 2.4. Ethics

Ethical approval was given by [Faculty of the Research Ethics Committee Faculty of Health Sciences University of Pretoria]. Institutional consent to use the staff in the two hospitals was obtained. Participants were required to read an information leaflet. Participants consented to participate in the study by completing the instrument items.

### 2.5. Preparing for Data Collection

This validation study was preceded by four steps. The first step was a concept analysis to determine the four attributes and definition of person-centred teamwork [12]. Step 2 involved an e-Delphi study with an international panel of experts to obtain a consensus on the four attributes and definitions [24]. The consensus definition for person-centred teamwork was “person-centred teamwork is a dynamic approach where the team, person(s) delivering care and person(s) receiving care, develop trust, and connectedness to meet the healthcare needs of the person. Underpinned in synergy, inclusivity and healthful relationships, the members of the team recognize the uniqueness of each individual, allowing mutual flourishing in striving to attain optimal outcomes [24].

Once consensus was reached, Step 3 included generating items to measure the concept of person-centred teamwork. The items were generated through a methodological literature search with the assistance of an information specialist. We first identified existing instruments on person-centredness and teamwork to identify relevant items. A total of 129 items were identified. The items were analysed and all doubles were removed. Then, the items were analysed by the two researchers for items that were similar in nature. This was done by means of an online discussion between the researchers. Once all the doubles and the items with similarities were sorted, the items were again sorted under the four attributes of person-centred teamwork. The item had to be indicative of the attribute it was under. The items were then further reduced in this process by indicating the subconstructs under each attribute. This revealed further similarities between items. This process of eliminating doubles and similar items was done five times. Once the item reduction was deemed to be complete, the items were then rephrased and sentence construction adapted to fit to the new instrument question. The rephrasing and sentence construction was repeated three times. Fifty eight items remained and was then discussed by the researchers. Items were moved to the correct attribute, and similar items were removed. The final number of items to be given to the participants were 43. Step 4 involved a Delphi study with a panel of international experts to reach consensus on the items that should be included in the instrument. A consensus was reached on 38 items to be included in the instrument [25].

A pilot study was also conducted. A sample of healthcare workers meeting the inclusion criteria was emailed and asked to participate. The purpose of the pilot study was to assess the clarity, understandability and functionality of each item. The instrument included Section A, which included biographical information, and section B, which included the 38 items. There were four constructs: Healthful Relations (HRs) and Recognition of the Uniqueness of the Individual (RUI), each with nine items; Inclusivity (INC) had six items and Synergy (SYN) had 14 items. The items were scrambled and not presented under each construct.

Fifteen healthcare workers were invited to participate voluntarily. Volunteers were emailed the instructions to either (1) print the instrument and complete it or (2) click on a Google form link and complete the instrument electronically. The participants were also asked if they clearly understood the items and if they recommended any appropriate changes. At the end of the questionnaire, there were three input questions: (1) Are the instructions to the instrument clear and understandable? (2) Is the layout of the instrument easy to use and functional? and (3) Could you easily understand the wording used? The participants were required to respond to these three questions. Participants were asked to provide feedback within two weeks.

Seven healthcare workers volunteered (response rate = 46%). The participants included nurses (*n* = 4), physiotherapists (*n* = 2) and dieticians (*n* = 1). Two participants were from the public hospital and five were from the private hospital. The participants' years of experience ranged from 5 to 33 years, with a mean of 17.14 and a standard deviation of 10.36. No changes to the layout, instructions or wording of the items were suggested. The same instrument was used during the validation. The data collected during the pilot study were not used for validation.

### 2.6. Population and Sample

The target population included healthcare workers who worked in the hospital setting. The sample was done on healthcare workers at two selected hospitals. Total sampling was used to select healthcare workers who (1) were nurses, medical doctors, dieticians or physiotherapists; (2) were directly involved in patient care and (3) were employed full time at one of the selected hospitals.

Various authors suggest different sample sizes when validating an instrument. Boateng et al. [26] suggested a sample size of 10 participants per item. Clark and Watson [27] suggested a sample size that is independent of the item number, namely, 300 participants. MacCullum et al. [28] suggested that a sample of 100 is poor, 200 is fair, 300 is good, 500 is very good and 1000 is excellent. We aimed to obtain 380 participants because the instrument had 38 items, excluding the items focussing on biographical information, which is in line with the recommendations of MacCullum et al. [28] and Boateng et al. [26].

### 2.7. Data Collection

The data were collected in two ways, namely, paper based and electronic based. The paper-based instrument consisted of four pages, where the first page included a description of the study and the researcher, the second page included the biographical information items (5 items) and the third and fourth pages included the 38 items measuring the perceptions of person-centred teamwork. The second data collection option involved an electronic link created on Google Forms that was sent to participants via e-mail or WhatsApp. The link took the participants to a page with the same information as the paper-based instrument. Once the participant clicks “submit,” the data are captured and stored. The data were collected by visiting each unit in the respective hospitals. The study was explained to the manager and staff present in the unit. The participants were given instructions to the manager to distribute the instruments to the staff. An envelope or closed box was used to ensure that the completed instruments were returned. We visited the respective units three times a week for 3 months. The completed instruments were then collected, and additional instruments were supplied. The electronic link was also given to the managers of the different groups. The manager then distributed the link to the staff by using WhatsApp or email. We captured the paper-based data in the electronic version. The data were subsequently exported to an Excel spreadsheet.

### 2.8. Data Analysis

The data were analysed using the software IBM SPSS Statistics Version 28 and RStudio 2023.06.0. The demographic characteristics were descriptively analysed. Exploratory factor analysis was not performed because the items were rigorously identified and validated in a Delphi study. The validity of the 38 items was assessed via a confirmatory factor analysis (CFA) using techniques that included the chi-square test of exact fit (1–5 acceptable), the comparative fit index (> 0.90 acceptable), the Tucker–Lewis index (> 0.90 acceptable), the root mean square error of approximation (RMSEA, < 0.07 indicates good fit) and the standardized root mean square residual (0-1 good fit) to measure item fit [26]. Factor loading was determined, and the heterotrait–monotrait (HTMT) ratio was used to determine discriminant validity (discriminant validity was confirmed when the HTMT was < 0.90). Bifactor analysis was used to determine general factor loading through chi-square tests, the comparative fit index, the Tucker–Lewis index, the RMSEA and the standardized root mean square residual. Bifactor indices were determined. To assess internal consistency, interitem correlations were examined, and the reliability coefficient was calculated by using Cronbach's *α*.

## 3. Results

### 3.1. Biographical Data

The data were collected from January 31 to March 31, 2023. A total of 388 participants participated, of whom 160 (41.2%) were from a private hospital and 228 (58.8%) were from a public hospital. The total population of the private hospital was *N* = 343, and the response rate was 46.5%. The public hospital instrument was distributed to *N* = 600 staff members (*n* = 228 responses were received), which is a 38% response rate.

Participants were from 138 wards; 136 participants were from intensive or high care units, 15 participants were from the theatre, 40 participants were from emergency departments and 48 participants from multiple units, largely from non-nursing categories. The professions that participated were 13 dietician (3.35%), 48 enrolled nurse (12.37%), 30 enrolled nursing assistants (7.73%), 19 medical doctor (4.89%), 32 physiotherapist (8.24%) and 238 registered nurses (61.34%). There were 8 participants who indicated “Other” under the question related to the ward where they worked. The category “Other” represented nursing students, medical doctor interns, nursing managers and administrators working in the ward. The years of experience in the participants' chosen profession ranged from 1 to 44 years, with most of the participants having between 10 and 25 years of experience.

### 3.2. Dimensionality

The dimensionality of the items was tested to determine whether the items were duplicated across two samples via CFA [26, 29]. CFA indicates the measurement of the model and the item fit indices. The item fit indices used were as follows: chi-square test of model fit, 2283.189; degrees of freedom, 655; chi-square/d*f*, 3.48 and *p* value, 0.001. The comparative fit index was 0.988, the Tucker–Lewis index was 0.987, the RMSEA was 0.080, the upper tier RMSEA was 0.084 and the standardized root mean square residual was 0.069 [26, 29]. The fit indices indicate a respectable item fit, in which the items are significant and the constructs are well measured. In addition, factor loading for all the constructs was determined. The four constructs had factor loadings on all items between 0.518 and 0.816. Discriminant validity was not achieved because the HTMT ratio ranged between 0.984 and 1.0. Therefore, discriminant validity was not achieved but rather the results indicated the presence of a general factor, which led to bifactor analysis. According to our bifactor analysis, the chi-square was 1.056, the comparative fit index was 0.999 and the Tucker–Lewis index was 0.999. All the items were used to measure the general factor.  Table 1 indicates the factor loading and mean scores of the person-centred teamwork instrument.

The following bifactor statistics were calculated for the omega (*ω*), omega subscale (ω_*s*_), omega hierarchical (ω_*H*_) and omega hierarchical subscales (ω_*HS*_) [30] to assess the unidimensionality of the general factor scale. The omega for the general score is (*ω* = 0.968), which implies that 96.8% of the variation in the total score can be attributed to common variance across the factors and that 3.2% of the variance is due to errors. For the subscales, the omegas were for HR (ω_*s*_ = 0.888), INC (ω_*s*_ = 0.832), RUI (ω_*s*_ = 0.848) and SYN (ω_*s*_ = 0.926). To assess the proportion of the variance in the general score, the omega *H* was calculated. The omega *H* for the general score is (ω_*H*_=.960), which implies that 99.2% (0.960/968 = 0.992) of the variance is attributed to the general factor, whereas 0.8% (0.008/968 = 0.008) of the variance is attributed to the factors. The omega *Hs* for the subscales were HR (ω_*HS*_ = 0.000), INC (ω_*HS*_ = 0.042), RUI (ω_*HS*_ = 0.050) and SYN (ω_*HS*_ = 0.028). The explained common variance (ECV) by the general factor was 0.864, which implies that 86.4% of the common variance is explained by the general factor and that 13.6% of the variance is explained by the four subscales. When omega is greater than 0.80, the general score is considered unidimensional [30]. The authors thus confirmed construct validity.

### 3.3. Reliability

Reliability was tested using Cronbach's *α*. A Cronbach's *α* < 0.70 indicates that the item consistently measures the construct [26]. The unidimensional reliability of all four constructs across all items had a Cronbach's *α* between 0.811 and 0.922. The reliability of each item indicates that the item contributes to instrument reliability.  Table 2 indicates the unidimensional reliability of the four constructs.

## 4. Discussion

This study describes the validation of an instrument to measure person-centred teamwork from the perspective of healthcare workers. The instrument was validated for use in healthcare settings. The sample of participants is representative of the healthcare population biographic data with the registered nurses that participated in the study being of a higher percentage than the national average [31]. The difference could be related to the private hospitals policy to employ more registered nurses in all units.

The data were collected using two methods, an electronic link and a paper-based instrument. The response rate on the electronic platform was low, with an average response rate between the two facilities of 15%, which is congruent with the findings of Wu et al. [32]. The paper-based platform response rate was 53% between the two facilities, which is considered an average response [32]. Keeping the response rate in mind, the aim was to have a sample size of 380 or more [26]. A good sample was obtained with 388 responses.

CFA was used to validate each item. The CFA yielded an excellent model fit and reliability of the factors. The instrument development process was driven by the theory of CFA [14, 33]. CFA indicates the good internal structure of the items used to measure the constructs, and the relationships between the items are consistent [26]. See Figure 1 for the measurement model of the four constructs: HR, RUI, INC, and SYN. The four constructs each had factor loadings with significant *p* values. The HTMT ratio confirmed that overlap occurred and that discriminant validity was not achieved. Therefore, bifactor analysis was used to evaluate the unidimensional construct while recognizing the multidimensionality of each construct [26]. Each item was assessed and found to measure a general factor. This high indication of reliability from the Cronbach's alpha indicated that all the items measured the concept of person-centred teamwork. Therefore, none of the items were reduced. The 38 items accurately measured person-centred teamwork during the initial testing. Similarly, the four constructs of the instrument accurately measured person-centred teamwork. The person-centred teamwork instrument will, therefore, enable the measurement of effective implementation of person-centred teamwork or allow for effective benchmarking for future interventions that aim to improve person-centred teamwork. The instrument can be used to monitor person-centred teamwork over time, offering empirical support for evaluating revisions aimed at implementing person-centred teamwork in practice. The use of all healthcare workers in testing the instrument further enhances its comprehensive applicability. Consequently, this instrument is envisaged to aid in measuring person-centred teamwork across all healthcare workers.

## 5. Limitations

This study proposes an instrument to measure the perceptions of healthcare workers about person-centred teamwork. The limitations of this study were as follows: (1) the instrument was validated in South Africa; therefore, the instrument should be further validated in other countries, (2) we did not request feedback about the time spent completing the instrument and (3) not using a random method for sample selection is one of the limitations of the research.

## 6. Conclusion

The person-centred teamwork instrument was developed and validated and found to be a reliable multidimensional scale comprising 38 items. The CFA indicated good model fit and reliability, with all four constructs exhibiting factor loadings confirmed via the HTMT ratio, indicating overlap. However, discriminant validity was not achieved, and the bifactor analysis affirmed the multidimensionality of each construct. The Cronbach's *α* values confirmed item reliability. The instrument facilitates the generation of evidence regarding the implementation of person-centred teamwork in practice. This information can be used to identify the developmental needs of person-centred teamwork in practice. The person-centred teamwork instrument may allow for comparative studies across various clinical and geographic settings.

## Figures and Tables

**Table 1:** The factor loading and mean scores of the person-centred teamwork instrument (final 38 items).

Item	Mean	Est	SE
I experience positive role modelling for the development of healthful relationships within the healthcare team	3.106	0.177	0.054
The team leader is sensitive to the needs of all team members	2.972	0.22	0.065
There is an effort to support and help each team member	3.106	0.097	0.063
Team members work collaboratively to agree on goals	3.054	0.07	0.05
Team members are encouraged to discuss what is important to them, as part of the team	2.979	0.075	0.074
Team members actively try to understand each other's perspectives	2.923	0.018	0.065
With the person receiving care's approval, their significant others are encouraged to actively engaged in the care received.	3.041	0.082	0.067
I feel acknowledged as a person within the healthcare team	3.021	0.038	0.076
Team members are encouraged to reflect on their practice within the team	2.974	0.021	0.056
When working with a person receiving care, language that they understand is used	3.211	0.023	0.049
Inputs from the person receiving care is valued by members of the healthcare team	3.222	0.109	0.059
Healthcare team members are encouraged to ask for help without being judged	3.186	0.008	0.051
Team members have developed shared values and beliefs	2.956	0.075	0.063
Facilitated reflection is used to develop practice according to agreed evidence	2.943	0.033	0.057
The healthcare team's achievements are celebrated	2.794	0.133	0.072
There is trust among the team members	2.755	0.072	0.07
Team members work collaboratively to resolve conflicts through shared decision-making	2.832	0.032	0.053
Healthcare team members listen to persons receiving care to identify needs, hopes and desires	3.191	0.258	0.068
The healthcare team is focused on their commitment to deliver individualized holistic care	3.162	0.438	0.108
Team members collaborate by agreeing to solutions for individualized care plans	3.075	0.328	0.119
Each team member has the freedom to be authentic within the team's values	2.948	0.27	0.0103
Care plans are discussed between the healthcare team, significant others and person receiving care	2.928	0.186	0.08
Each team member's contribution is valued	2.995	0.535	0.278
Each team member's knowledge, skill and expertise are respected and valued	3.013	0.293	0.156
Conflict within the team is managed by the team without affecting care provided	2.987	0.187	0.068
Healthcare team members discuss care plans to ensure consistency of practice	2.992	0.129	0.061
Conflict within the team is managed by team members without affecting the team cohesion	2.887	0.18	0.066
Each team member's contribution is acknowledged and valued	2.889	0.102	0.051
The healthcare team is able to reach consensus on areas of disagreement	2.897	0.066	0.051
Where the person receiving care has capacity, s/he is involved in decision-making processes	3.162	0.105	0.063
Decision-making process includes the person receiving care's significant others, where appropriate.	3.17	0.095	0.075
Practices inconsistent with the healthcare team's shared values and beliefs are challenged	3.054	0.231	0.064
Healthcare team members collaborate to provide best practice	3.198	0.23	0.065
Team effectiveness is evaluated by the person(s) receiving care	3.031	0.17	0.066
Communication (verbal and nonverbal) between team members occurs in a respectful manner	2.974	0.048	0.054
Care of the person receiving care, is effectively organized and communicated	3.137	0.16	0.057
Team effectiveness is evaluated by the team	2.961	0.196	0.066
I am respected by the team	3.113	0.073	0.064

**Table 2:** Unidimensional reliability of the four person-centred teamwork instrument constructs.

Unidimensional reliability				
Estimate	Healthful relations	Recognizing uniqueness individual	Inclusivity	Synergy
	Cronbach's *α*	Cronbach's *α*	Cronbach's *α*	Cronbach's *α*
Point estimate	0.872	0.859	0.811	0.922
95% CI lower bound	0.852	0.837	0.779	0.910
95% CI upper bound	0.890	0.879	0.838	0.933

Abbreviation: CI = confidence interval.

## Data Availability

The data used to support the findings of this study are available on request from the corresponding author.
